# Snapshot of narcotic drugs and psychoactive substances in Kuwait: analysis of illicit drugs use in Kuwait from 2015 to 2018

**DOI:** 10.1186/s12889-021-10705-z

**Published:** 2021-04-07

**Authors:** Abdullah Al-Matrouk, Mohammed Al-Hasan, Husain Naqi, Neamat Al-Abkal, Hanan Mohammed, Meshaal Haider, Dalal Al-Shammeri, Haider Bojbarah

**Affiliations:** 1 Narcotic and Psychotropic Laboratory, General Department of Criminal Evidence, Ministry of Interior, Al-Dhajeej, Block 1, Al-Farwaniya City, Kuwait; 2 Toxicology Laboratory, Department of Criminal Evidence, Ministry of Interior, Farwaniya city, Kuwait; 3 Kuwait Police Academy for Security Sciences, Ministry of Interior, Al-Farwaniya City, Kuwait

**Keywords:** MENA, Kuwait, GC-MS, LC-MS-MS, Illicit drugs, Psychoactive substances

## Abstract

**Background:**

The misuse of illicit substances is associated with increased morbidity and mortality; thus, substance abuse is a global health concern. The Arabian Gulf region is considered a crossing point and a consumer of illicit drugs. However, a lack of laboratory-based research has limited the scientific assessment of drug misuse in the Arabian Gulf region. Thus, an up-to-date analytical representation of the drug situation is warranted.

**Methods:**

We investigated the type and quantity of detained narcotic drugs and psychotropic substances from 2015 to 2018, representing a population of approximately 4 million people, in addition to the number of abusers and mortality among abusers. In total, 6220 cases from the Narcotic and Psychotropic Laboratory and 17,755 cases from the Forensic Toxicology Laboratory were reviewed and analyzed. Substances were identified and documented using gas chromatography–mass spectrometry and liquid chromatography–mass spectrometry.

**Results:**

Cannabis, including marijuana, was the most seized substance, followed by heroin, opium, and cocaine. Amphetamines, including methamphetamine, in the form of powder or pills, were seized in larger quantities than other psychoactive substances. The most consumed substances were, in order, amphetamines (including methamphetamine), benzodiazepines, cannabis, and heroin. We identify the common drugs in postmortem specimens, according to sex, from suspected drug-related deaths. The most common single drug identified were heroin, benzodiazepines, and methamphetamine. Similarly, the multiple-drug cocktail of heroin–benzodiazepines, cannabis–benzodiazepines, and cannabis–amphetamines, were detected frequently.

**Conclusions:**

The data shows that cannabis is the leading type of illicit substance seized. Deaths resulting from benzodiazepines and heroin abuse were the highest in the single drug category, while heroin-benzodiazepines combination deaths were the highest in the multiple-drug category. Methamphetamine was the most abused illicit drug in Kuwait. These findings revealed the illicit drug abuse situation in the State of Kuwait, in a region that suffers from the scarcity of information regarding illicit substances. Thus, providing valuable information for drug enforcement, forensic analyst, health workers on national and international levels.

**Supplementary Information:**

The online version contains supplementary material available at 10.1186/s12889-021-10705-z.

## Background

The use of illicit drugs is a global concern. These substances for which extra-medical uses have been prohibited and criminalized under international drug control treaties [1]. Despite international efforts to eliminate drug abuse, the global consumption trend of these substances increases continuously. In 2018, it was estimated that approximately 275 million people (~ 5.5% of the world’s population) had used illicit substances at least once, which is an increase of 30% compared with that in 2009 [2, 3]. Thus, extra measures are needed to tackle this ongoing crisis.

Illicit substance abuse imposes enormous costs on the global health and economy [4]. The use of these substances carries risks of adverse health outcomes. Drug abuse is accountable for roughly 1.5% of the global disease burden and was responsible for more than 750,000 premature deaths in 2017 [3]. The use of these substances increases the risks of adverse health conditions, including but not limited to disability, viral infections, sepsis, thrombosis, and endocarditis [5]. Thus, the abuse of illicit drugs adds a severe financial burden on the global economy. The International Narcotics Control Board [6] estimates that the cost of medical care associated with drug misuse is more than $200 billion annually.

The global market for drug trafficking continues to grow each year. The term “drug trafficking” refers to the cultivation, production, distribution, and sale of illicit substances. In 2017, the United Nations Office on Drugs and Crime (UNODC) estimated that the global market for drug trafficking is worth $426 to $652 billion [7].

The Middle East and North Africa (MENA) are considered significant areas for illicit substance trafficking. In terms of production, cannabis (Morocco and Lebanon), opiates (Egypt), Khat (Yemen to Somalia), and amphetamine-type stimulants (ATS) (Egypt and Lebanon) are the most produced substances [8]. Owing to its geographical location situated between different trafficking routes and its widespread borders, MENA is recognized as a major transit area for illicit substances. Indeed, MENA links the major drug producers to the vast markets in Europe and the Gulf countries. Illicit substances that flow through MENA include opiates (Golden Crescent to Europe), cannabis (Morocco to the Gulf Countries), cocaine (Latin America to Europe), and amphetamine-type stimulants (Western Africa to Europe) [9]. Because of its geographical location being a passage between continents, drug addiction and abuse in MENA countries are high. As a result, legislative authorities in these countries have imposed harsh penalties for drug-related offenses to restrict and reduce the epidemic of recreational use of illicit drugs. However, such legislation only made illegal drug trafficking more lucrative [10].

Despite the importance of this topic, to our knowledge, the few studies published thus far concerning illicit substances that have been seized or consumed, or the associated-death rates, in the Middle East in general, and more specifically in Kuwait (representing a population of approximately 4 million people), have not been comprehensive [11, 12]. Additionally, most studies that have been previously conducted in Kuwait were outdated survey-based studies that did not rely on solid analytical data generated by laboratory-based experiments [13, 14]. Moreover, the only previous analytical-based study is outdated [15].

A cross-sectional questionnaire conducted on Kuwaiti male students (*n* = 1587) reported that the most commonly used illicit substances were marijuana (11%), stimulants (7.1%), cocaine (2.2%), and heroin (1.3%) (13). Omu et al. (2017) performed a survey on illicit drug abuse among teenagers (15 to 18 years) between September 2012 and June 2013 [14]. The results showed that the trend of illegal substances abuse is increasing, especially among older teenagers. Hashish (marijuana) was the most commonly used illicit substance among both current users and previous users, 3.7 and 5.3%, respectively [14]. A Kuwaiti pilot study confirmed the association between levels of self-esteem and anxiety with drug user behavior [16]. Wilby and Wilbur assessed narcotic requirements in different Arabic-speaking countries in the Middle East region [17]. Bahrain and Kuwait had the highest estimated rate (364 and 352 defined doses per million inhabitants per day, respectively), as well as the highest rates of growth (2008–2012).

Radovanovic et al. assessed the prevalence and trend of different psychoactive substances in Kuwait from 1992 to 1997 by screening 3781 biological samples [15]. According to the study, the most used substances were as follows: cannabinoids, benzodiazepines, opiate, and amphetamine. Other drugs were also identified but at insignificant levels; these included methadone, cocaine, and phencyclidine [15]. However, the information gained in that study was based only on a preliminary analysis (screening tests) of biological samples and is now outdated. Consequently, the current study is aimed to identify the types of narcotic drugs and psychotropic substances that were seized and consumed in Kuwait from 2015 to 2018. This was achieved using a Mass Spectrometric (MS) based approach for the analysis of drug (6220) and toxicological (17755) samples, providing a clear picture on the recent drug situation in Kuwait which provide local, regional and international public health workers and forensic analysts with data that are normally scarce in this region.

## Methods

### Narcotics and psychotropic laboratory (NPL) procedures

Receipt of Seized Narcotic Drugs and Psychotropic Substances.

**Fig. 1:**
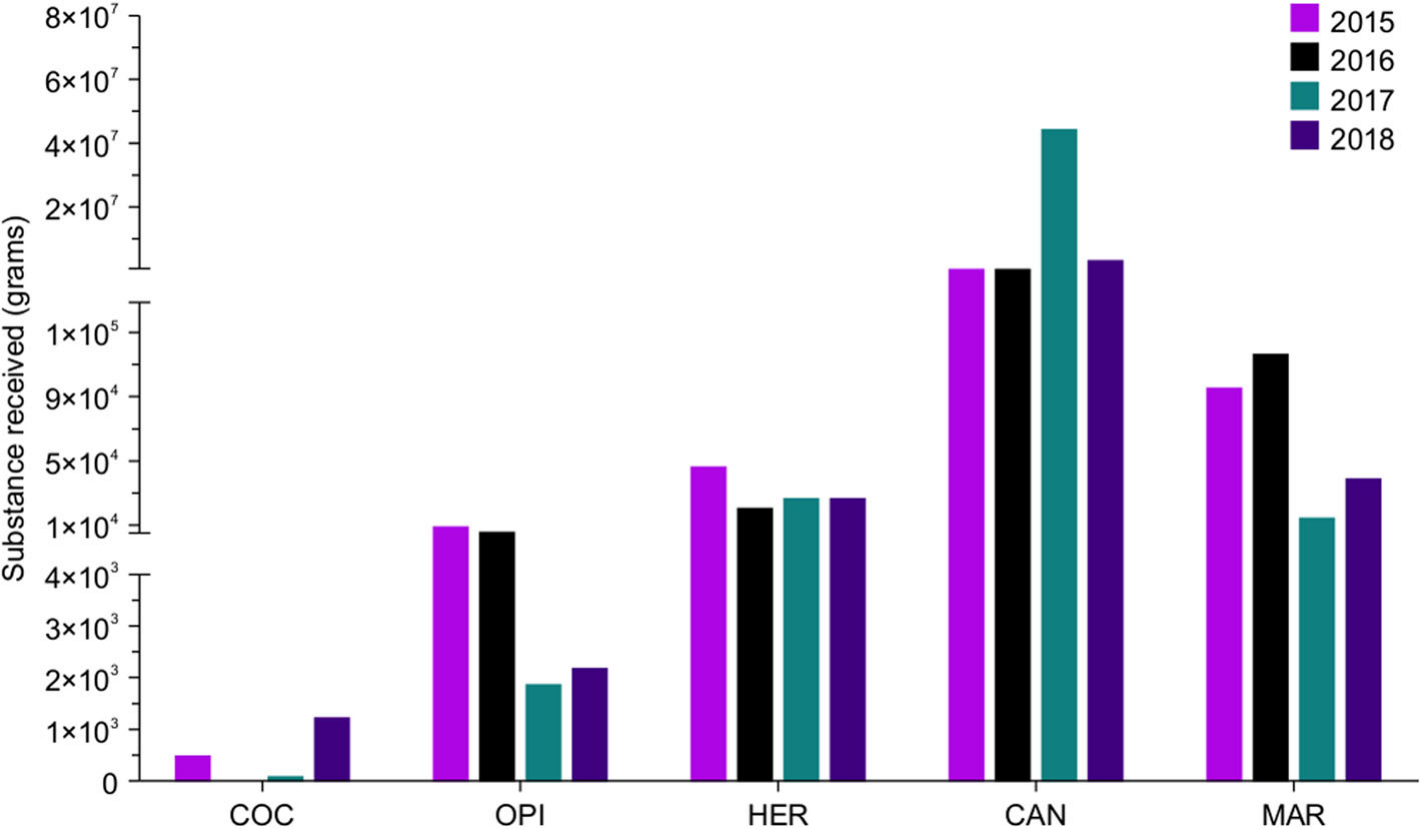
Quantities of different narcotic drugs (grams) received by NPL in Kuwait during 2015–2018; each year is depicted in a different color. COC, cocaine; OPI, opium; HER, heroin; CAN, cannabis; MAR, marijuana

**Fig. 2:**
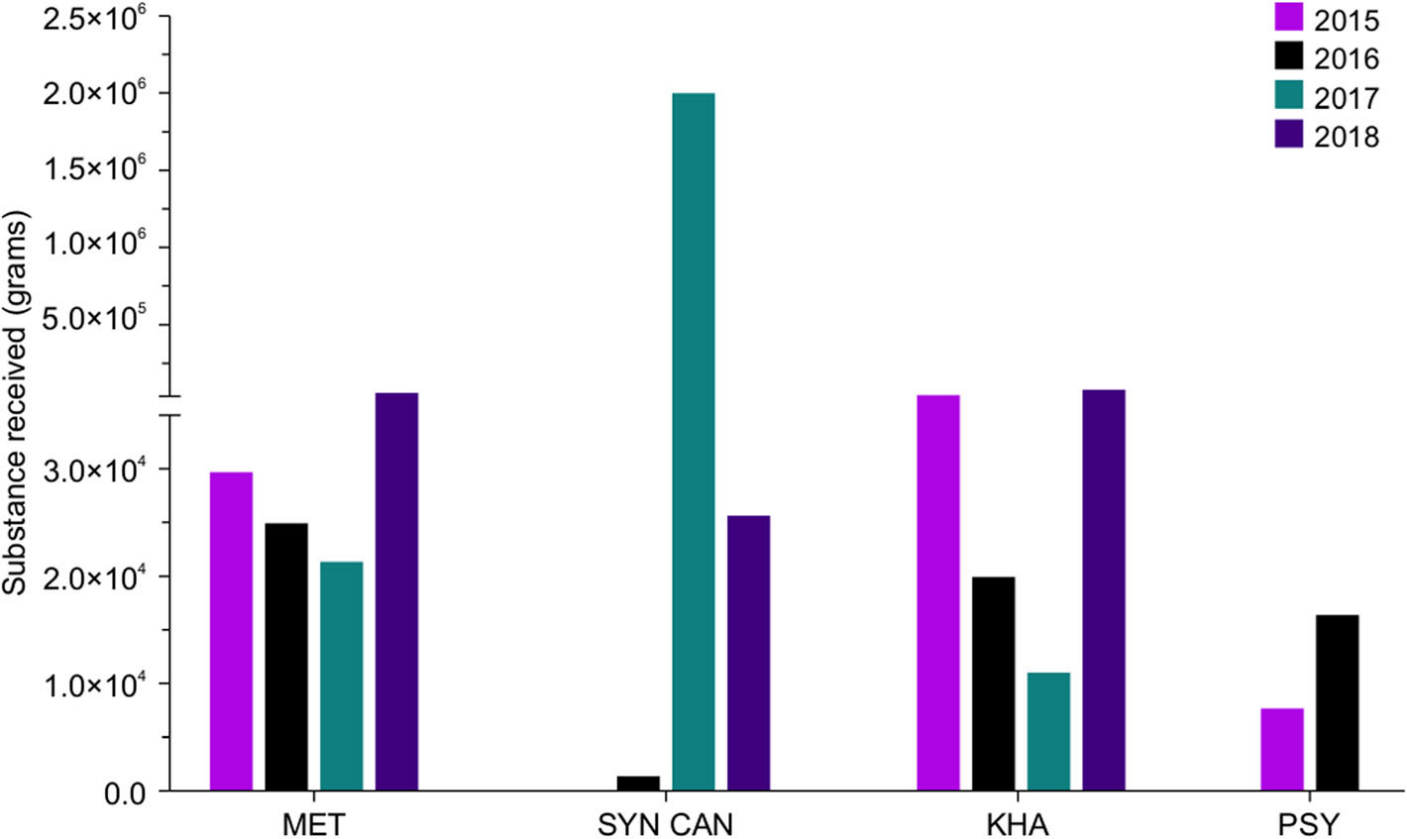
Bar chart showing quantities of psychoactive substances seized in Kuwait during 2015–2018. Each year is depicted in a different color. MET, methamphetamine; SYN CAN, synthetic cannabinoids; KHA, Khat; PSY, psilocybin mushrooms

**Fig. 3:**
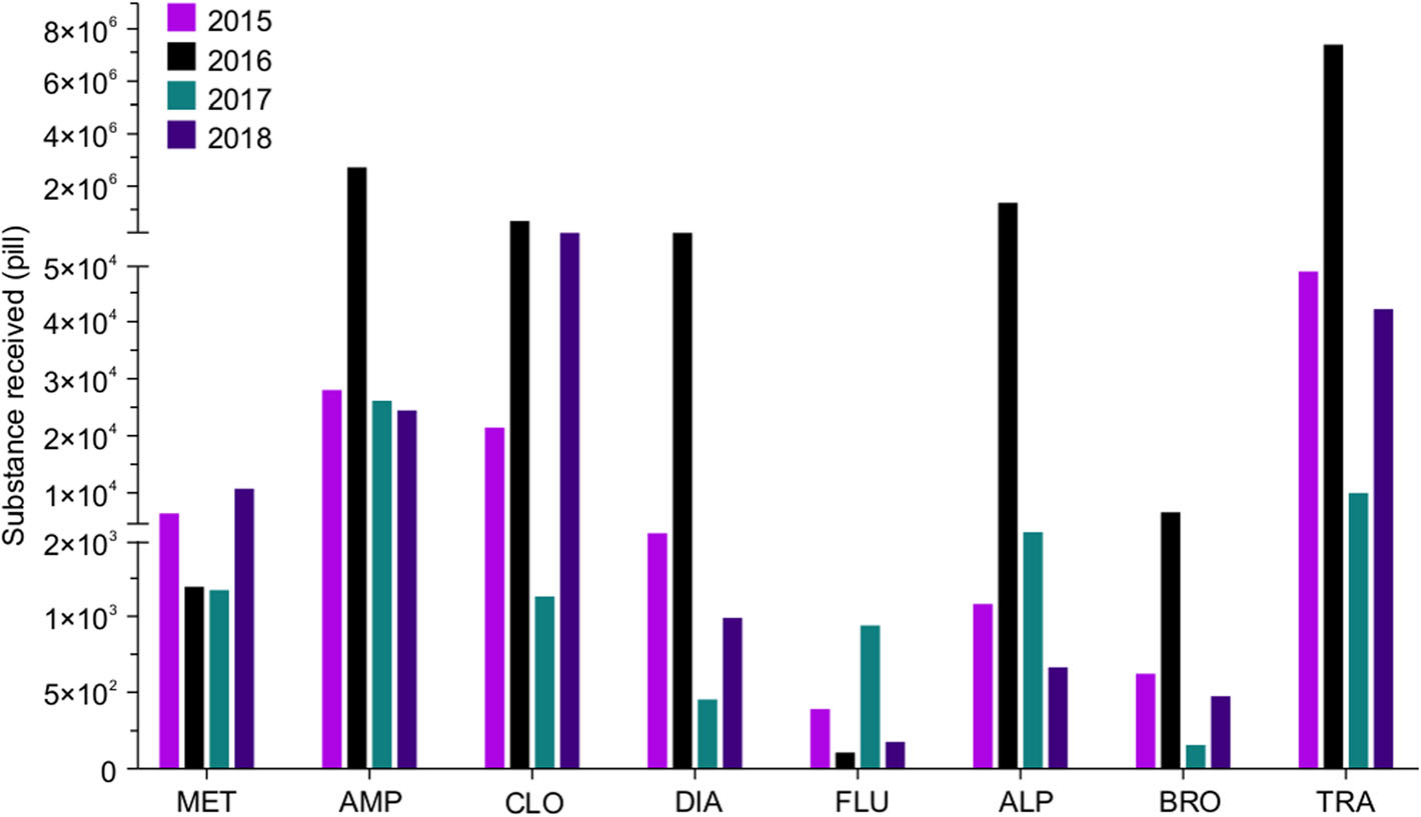
Quantities of seized psychoactive pills (number of pills or capsules) in Kuwait during 2015–2018. Each year is depicted in a different color. MET, Methamphetamine; AMP, Amphetamine; CLO, Clonazepam; DIA, Diazepam; FLU, Flunitrazepam; ALP, Alprazolam; BRO, Bromazepam; TRA, Tramadol. The y-axis is discontinuous at two different points due to the large variation in data

We have divided the received materials into two groups: (1) substances that can be weighed and thus reported in grams, including liquid materials (i.e. cannabis oil), powders (i.e. heroin and cocaine), vegetal materials (i.e. marijuana and damiana leaves containing synthetic cannabinoids), and solid blocks (such as cannabis and opium blocks), and (2) substances that can be counted, such as pills and capsules (i.e. tramadol, clonazepam, diazepam and alprazolam). Narcotic substances that were received by the NPL and are scheduled and documented in this report include cocaine, opium, heroin, cannabis (resin, oil and hashish), and marijuana (Fig. 1). Psychotropic substances that were received by the NPL and scheduled and documented in this report include methamphetamine, synthetic cannabinoids, Khat, and psilocybin mushrooms, which can all be weighed in grams (Fig. 2), and methamphetamine, amphetamine, tramadol, and benzodiazepines (such as clonazepam, diazepam, flunitrazepam, alprazolam, and bromazepam), which can all be counted as pills or capsules (Fig. 3). Photographic images were taken as representative examples for some of the seized substances (Additional file 1).

Seized narcotic drugs and psychotropic substances were received by the Narcotic and Psychotropic Laboratory (NPL) of Kuwait, a division of the Forensic Laboratories, General Department of Criminal Evidence. NPL is the only accredited laboratory in Kuwait that conducts drug-related investigations and routine drug testing to provide expert opinion (reports) for the court of law*.* Seized materials were sent to the NPL by several governmental agencies. Additionally, drug samples brought in for analysis included details of the origin of the sample e.g. place of seizure, date, physical appearance, name of suspect. Toxicological samples data included the type of sample (blood, urine), date, name of suspect.

The current study reviews and analyses documented cases that were received by the NPL and the Forensic Toxicology Laboratory (FTL) from January 2015 to December 2018. It also highlights the protocols and the experimental procedures that were used by the laboratory specialists at the NPL to generate the reports. Only reports that were generated from cases that were positive for the presence of at least one illicit drug (i.e., those listed in the schedules of narcotic drugs or psychotropic substances) were reviewed and analyzed.

### Processing seized materials in the NPL

All procedures were performed according to the required legal provisions and the chain of custody. Regarding the analysis of seized drugs, we followed the recommendations of the Scientific Working Group for the Analysis of Seized Drugs (SWGDRUG) [18]. The mission of SWGDRUG is to recommend minimum standards for the forensic examination of seized drugs and to seek the international acceptance of these standards [18].

### Color (spot) tests

Color (spot) is the most commonly used presumptive screening test in forensic laboratories. Specific instructions and protocols described by *Clarke’s Analysis of Drugs and Poisons* were used for the identification of the illicit drugs [19]. Marquis reagent was used for the detection of Opiates amphetamine, and methamphetamine, Duquenois-Levine and Fast Blue B reagent used for testing for cannabinoids in cannabis, and Scott’s reagent for the detection of cocaine.

### Macroscopic and microscopic examination for Cannabis

Macroscopic analysis (visual characterization) was used to document different cannabis species. The microscopic examination of leafy materials was performed using a stereomicroscope (Stemi DV4, Carl Zeiss, Jena, Germany), equipped with a cold light (Zeiss KL1500 LCD, Jena, Germany). Analysis included the identification of botanical characteristics such as cytolithic hairs (bear claw appearance), elongated hairs on the underside of the leaf, and resin glands (glandular hair).

### Thin layer chromatography (TLC)

TLC was performed on pre-coated aluminum TLC-sheets (20 × 20), with a 0.25-mm silica gel layer thickness. Solid samples were dissolved in methanol (MeOH; HiPerSolv CHROMANORM, HPLC grade, BDH prolabo) (VWR International, Fonenay-sous-Bois, France) then spotted onto a TLC plate using capillary tubes (Terumo corporation, Tokyo, Japan). The mobile phases were prepared and used according to the recommendation of *Clarke’s Analysis of Drugs and Poisons* [19]. Plates visualization was acheived using a short-wave ultraviolet (UV) light source and the ultraviolet fluorescent indicator ALUGRAM® Xtra SIL G SIL UV254 (Macherey-Nagel Gmbh, Duren, Germany). Rf values were calculated for each sample, for comparison with standards.

### Ultraviolet-visible spectroscopy (UV-Vis)

Ultraviolet-visible spectroscopy technique used to identify a number of different compounds, including ketamine hydrochloride, cocaine hydrochloride, diazepam, phenobarbital, and barbital [20]. In our laboratory, a Cary 60 UV-Vis spectrophotometer (Agilent Technologies, Santa Clara, CA, USA) was used for these measurements, and the spectra were recorded using the Cary WinUV Scan software (Agilent Technologies, Santa Clara, CA, USA).

### Attenuated Total reflection-Fourier transformed infrared (ATR-FTIR) spectroscopy

It is used as a confirmatory method for the detection of a variety of different drugs including benzodiazepines, amphetamine, methamphetamine, MDMA, lysergic acid diethylamide (LSD), cocaine, opium, heroin, morphine, synthetic cannabinoids, and cathinones. Most of the samples that were examined using this method in our laboratory are in a solid form. IR spectra were recorded using a Bruker ALPHA spectrometer (Bruker Optics, Ettlingen, Germany).

### Gas chromatography-mass spectrometry (GC-MS)

The method used for GC-MS analysis in this paper was adapted from our previous study [21]. GC-MS analysis was used exclusively for identification purposes in this study; no quantification was performed. Additionally, samples were analysed in triplicates (*n* = 3).

GC-MS vials were analyzed in a GC 7693 Gas Chromatograph (Agilent Technologies, Santa Clara, CA, USA) with an autosampler, and mass spectroscopy was performed using a 5977B GC/MSD Mass Selective Detector (Agilent Technologies, Santa Clara, CA, USA). GC-vials, GC-vial lids, and GC-vial inserts were also purchased from Agilent Technologies (Santa Clara, CA, USA).

The GC-MS parameters were set as reported in the methodology from our previous study [21]. The injection port temperature was set to 250 °C, the splitless injection volume was 2.0 μL under a purge flow of helium gas at 3 mL/min, and the solvent delay was set to 3 min. The wash steps were: four pre-injection washes, four post-injection washes, two sample washes, and six sample pumps. The initial temperature was set to 100 °C for 4 min. Ramp 1 was set to 10 °C/min until reaching 280 °C, where it remained for 2 min. The °C/min rate for Ramp 2 was set to 6 °C until it reached 300 °C, where it remained for 5 min. An HP-5MS UI column of 30 m length, 0.25 mm inner diameter, and 0.25 μm film thicknesses (Agilent, Waldbronn, Germany) was used with the flow rate set to 1 mL/min. The MS ionization mode was electron ionization (EI) set at − 70 eV, with an ion-source temperature of 280 °C and an interface temperature of 290 °C. Ions were monitored using SCAN mode. Cayman Spectral, FORCHEM, and NIST 14 Libraries were used for comparative analysis.

### Toxicology laboratory procedures

One of the job duties of the Forensic Toxicology Laboratory (FTL) in the General Department of Criminal Evidence is to analyze drug metabolites in biological matrices including urine and blood. Additionally, FTL is the only lab in the country authorized to analyse toxicological samples. Each toxicology analysis is then translated into an official report to confirm or deny drug abuse suspension, and to be used for subsequent legal actions.

This study only analyzes reports from specimens that yielded positive results for drug abuse. In addition, some positive cases were not reported herein, as the toxicants are irrelevant to the current study. All data were collected with permission from the Ministry of Justice and the Ministry of Interior.

### Urine sample collection from deceased cases

If available, urine was syringed from the bladder of deceased individuals, using a 10 mL syringe as soon as they were admitted to the Forensic Medicine Unit (FMU). The specimen was then stored at 20 °C until analysis. Living individuals (hospitalized or apprehended) provided 10 mL of urine samples in containers, which were sealed and taped to prevent adulteration.

### Blood sample collection

Venous blood was taken from a cubital vein by a physician or registered nurse of living or deceased individuals, using 10 mL gray-stopper evacuated glass tubes containing sodium fluoride (100 mg) and potassium oxalate (25 mg) as preservative agents.

### Screening of urine and blood samples

Urine samples were screened by Evidence® Drug of Abuse (DOA) Array I Urine Plus (DOA I URN P) assays (Randox, Crumlin, County Antrim, UK). Evidence® Drug of Abuse (DOA) The Ultra whole blood (DOA ULTRA WB) (Randox, Crumlin, County Antrim, UK) assay was used for semi-quantitative determination of the parent molecule and the metabolites of illicit drugs in human blood.

### Liquid chromatography with tandem mass spectrometry (LC–MS/MS)

Details of the LC-MS/MS method and the materials used have been published in detail in our previous publication and are outlined below [21]. Prior to analysis, solid phase extraction (SPE) was used to extract illicit drugs from urine and blood samples for testing. As with GC/MS analysis, samples were analysed in triplicates (*n* = 3).

CHROMABOND® C18 columns with a volume of 3 mL containing 500 mg sorbent (Macherey-Nagel, Düren, Germany) were used for solid-phase extraction of urine samples. The pH of the urine samples (10 mL) were adjusted according to Macherey-Nagel guidelines for specific drugs of abuse and samples were then centrifuged.

The compound database used for analysis was the Forensic Database (Forensic DB). However, the identification of the unknown or examined samples by the library database in this step was only tentative. Therefore, reference standards were used following preliminary identification of the unknown sample using the library, and an LC–MS/MS procedure was developed. The LC–MS/MS procedure used in the current study was a two-step scheme that was performed after injecting 10 mg/mL of the standard in methanol and 100 ng/mL of the standard in blank urine (for urine samples) or blank blood (for blood samples). The standards were extracted from blank urine or blank blood using the SPE method and used as quality controls.

### Data analyses

Raw data visualization, analysis, and graph creation were conducted using GraphPad Prism version 6 (Prism Software Corporation) and Microsoft Excel 2016 (Microsoft Corporation).

## Results

NPL received a total of 6220 drug abuse cases from 2015 to 2018. In terms of cases per year, 1832 cases were received in 2015: in 2016, 2017, and 2018, to 1506, 1356, and 1526 cases, respectively. For analytical purposes, the current study focused on cases with positive drug outcomes: the presence of at least one illicit drug listed in the narcotics or psychotropic substances schedules. Negative samples represented 20% of the total number of drug samples. Regarding the toxicological analyses, data for the analyzed and reviewed materials were collected from FTL archives (January 2015–December 2018). Regardless of the toxicological outcome (positive/negative), a total of 17,755 cases were received by the FTL (2015–2018). The numbers per calendar year were; 5171 in 2015, 3708 in 2016, 4115 in 2017, and 4761 in 2018.

### Quantities of narcotic drugs seized in Kuwait from 2015 to 2018

Figure 1 shows scheduled and documented raw narcotic substances received by the NPL between 2015 and 2018. These include cocaine, opium, heroin, cannabis, and marijuana. In here we will mention the amounts received per year for each of the substances and describe the amount of 10 largest samples, in order to allow identification of bias due to 1 or more large samples.

Overall, cocaine was the lowest received illicit raw material, initially 494.890 g received in 2015, 3.080 g in 2016, 105.840 g in 2017 and 1240.600 g was received in 2018. The 10 largest samples of cocaine were in the range of (0.256 g to 10.5 g) during these years. The quantities of seized marijuana were 95,681.730 g in 2015, 116,472.660 g in 2016, 14,614.133 g in 2017, and 39,809.860 g in 2018 and the 10 largest samples during these years were in the range of (10,200 g to 17.500 g). The quantity of cannabis was 652,759.480 g in 2015, 632,052.630 g in 2016, 43,781,809.500 g in 2017 and 3,234,105.988 g in 2018 with the 10 largest samples during these years are in the range of (10,300,657 g to 15,500,250 g). The quantity of opium (OPI) received to the NPL was 10,221.460 g in 2015, in 2016 it was 5677.320 g, 1868.060 g in 2017 and 2168.830 g in 2018 and the largest 10 samples were in the range of (1246 g to 3341 g). The quantity of seized heroin (HER) remained almost constant throughout the 4 years 47,567.860 g in 2015, 21,272.740 g in 2016, 26,485.288 g in 2017 and 25,960.135 g in 2018 (Fig. 1). The largest 10 samples received for heroine were in the range of)5013 g to 7346 g). A detailed list of the quantities of the received narcotic drugs (grams) is shown in Additional file 2.

### Physical characteristics of narcotic drugs seized in Kuwait from 2015 to 2018

While cocaine was received mostly as a white powder (95% of cases), in a few cases (5%) it was received in its smokable form (crack cocaine); that is, processed with sodium bicarbonate (baking soda) and water. Heroin was received as a powder with a color that ranges from light to dark brown (and, in very rare cases, off-white). Opium in all cases was received as a sticky black solid block with a distinctive odor. Cannabis was mostly received as blocks of various sizes, and sometimes as “shatter” (concentrated cannabis sheets) or in an edible form (candies, brownies, and cookies). In very rare cases, tetrahydrocannabinol (THC), which is the principle psychoactive constituent of cannabis, was detected in some of the received pills. Marijuana was mostly received in dried plant materials or in herb form, but in very rare cases the two plant types (*Cannabis sativa* or *indica*) were received.

### Amounts of psychotropic substances seized in Kuwait from 2015 to 2018

The total quantities of psychotropic substances (grams), including methamphetamine, synthetic cannabinoids, Khat, and psilocybin mushrooms are presented in Fig. 2. For the psychotropic substances, the quantity of methamphetamine (MET) was 29,732.920 g in 2015, 24,826.770 g in 2016, 21,376.662 g in 2017 and in 2018 it reached 60,082.84 g. The largest 10 samples were in the range of (10,967 g to 11,346 g). Similarly, the quantity of Khat (KHA) was 50,698.62 g in 2015, 19,885.200 g in 2016, 11,109.500 g in 2017 and 83,183.29 g in 2018. The largest 10 seized samples of Khat during these years were in the range of (3437 g to 8495 g). For synthetic cannabinoids (SYN CAN), no data was documented in 2015, and the quantity reported in 2016 was 1420.300 g. This is primarily because these substances were only listed and banned in October 2016. Thus, the data stated herein report the seized materials only after the banning legislation. In 2017, the quantity reached 2,003,897.784 g, and then the quantity became 25,561.66 g in 2018. It is believed that these changes are due the criminalization of synthetic cannabinoids, leading drug users/abusers to revert back to classical cannabinoids products. The 10 largest cases were in the range of (10,536 g to 13,438 g). Finally, psilocybin mushrooms (PSY) was only seized in 2015 with quantity of 7549.320 g and 2016 with quantity of 16,338.500 g (Fig. 2) and the quantities of the largest 10 cases were in the range of (2069 g to 3795 g). The exact seized quantities of psychoactive substances (grams) are listed in Additional file 3.

### Physical characteristics of psychoactive substances seized in Kuwait from 2015 to 2018

In the vast majority of cases, methamphetamine was received as crystal-like glass fragments or small, shiny, white rocks. In very few cases, seized amphetamine occurred in different colors (pink, light blue, and even dark blue). Methamphetamine was also seized in the form of tablets of different colors and sizes. However, the data shown in Fig. 2 do not include the tablet forms of methamphetamine. Descriptions of the physical characteristics of synthetic cannabinoids have been previously reviewed [21]. Khat usually comes in the form of dried plant materials or herbs, and the detected active stimulating compound in these materials are cathinone and/or cathine. Psilocin and/or psilocybin are the active psychoactive compounds in some mushrooms species (including *Psilocybe mexicana* and *P. cubensis*, formerly *Stropharia cubensis*) that are commonly known as magic mushrooms. NPL obtains these active compounds by extracting them from the mushroom plant except in the very rare cases that they are received as a liquid substance.

### Quantities of psychotropic tablets seized in Kuwait from 2015 to 2018

Figure 3 illustrates the quantities of seized psychoactive pills (number of pills or capsules) in Kuwait from 2015 to 2018. Most of the psychoactive substances are received in the form of pharmaceutical products, pills, or capsules. In 2015, approximately 6500 methamphetamine (MET) pills were seized. The quantity became almost 1000 pills in the following 2 years, though in 2018 it subsequently reached approximately 10,000 pills. Our data show that there was a high demand for amphetamine (AMP), tramadol (TRA), and clonazepam (CLO) in the drug market in Kuwait during the last 4 years. The quantity of seized amphetamine (AMP) remained almost the same during the 4 years of the study, except in 2016, in which the number reached 10,711.98 pills. The quantities of tramadol, clonazepam, diazepam (DIA), flunitrazepam (FLU), alprazolam (ALP), and bromazepam (BRO) fluctuated throughout the years. In comparison to the other psychoactive pills, FLU and BRO had a low demand in the Kuwaiti market. Additional file 4 shows the precise quantities of seized psychoactive (pills) in Kuwait from 2015 to 2018. The largest 10 cases were in the range of (5054 pills to 8086 pills) for all of the above mentioned tablets, during the 4 years of the study.

### Forensic toxicology laboratory

The Forensic Toxicology laboratory (FTL) received a total of 17,755 cases during the four-year period (2015–2018). The present study focused on positive test drug cases only. The LC-MS-MS data were obtained from the analysis of biological matrices (blood and urine) of living, or deceased individuals.

### Illicit drug abusers (male and female) in Kuwait from 2015 to 2018

Our results show that many different narcotic drugs and psychotropic substances were abused. These include methamphetamine (MET), amphetamine (AMP), benzodiazepines (BEN), cannabinoids (CAN), heroin (HER), tramadol (TRA), and cocaine (COC).

**Fig. 4:**
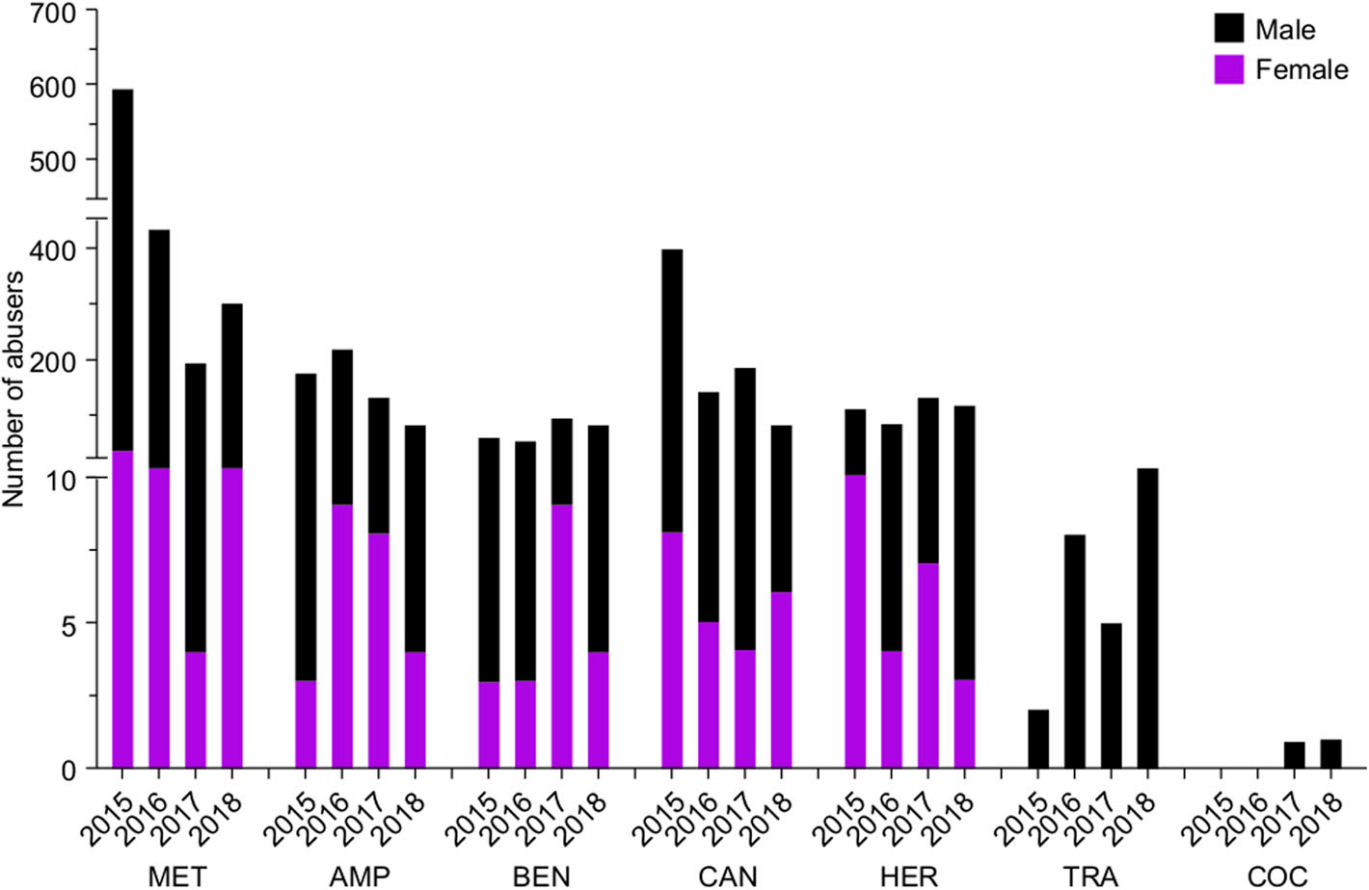
Bar chart showing the number of abusers of different illicit drugs during 2015–2018. Number of male abusers is depicted in black, whereas the number of female abusers is depicted in purple. MET, methamphetamine; AMP, amphetamine; BEN, benzodiazepine; CAN, cannabis; HER, heroin; TRA, tramadol; COC, cocaine. The y-axis is discontinuous at two different points due to the large variation in data

Our data shows that methamphetamine was the most abused illicit drug in Kuwait throughout 2015–2018, whereas cocaine had the least number of users. For the remaining illegal drugs, no dramatic changes were observed in terms of the number of abusers. Amphetamine, benzodiazepines, cannabis, and heroin were also highly abused. Moreover, the change in the number of abusers was inconsistent, and it fluctuated throughout the 4 years of study (Fig. 4).

**Fig. 5:**
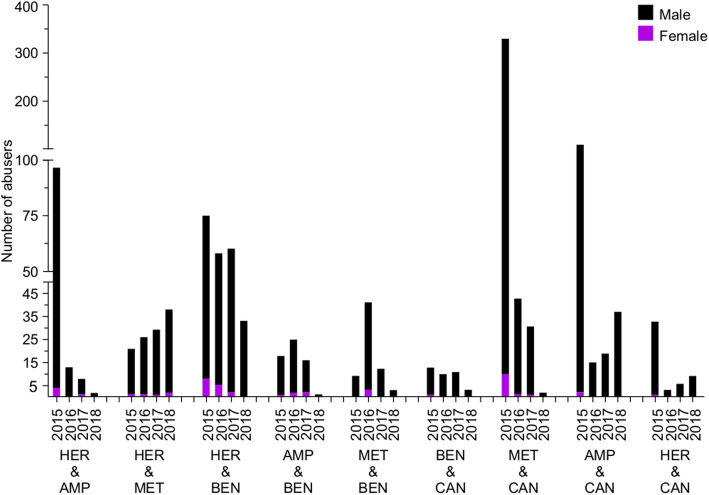
Bar chart showing abusers (number) of two different illicit drugs in Kuwait during 2015–2018. Male and female abusers are distinguished by black and purple colors, respectively. MET, methamphetamine; AMP, amphetamine; BEN, benzodiazepine; CAN, cannabis; HER, heroin. The y-axis is discontinuous at two different points due to the large variation in data

The abovementioned abused substances were found to be abused alongside other combinations of illicit drugs (Fig. 5). For instance, heroin was frequently abused, along with amphetamine, methamphetamine, and benzodiazepines. Cannabis was abused with amphetamine, benzodiazepines, methamphetamine, and heroin. All these abused combinations are shown in Fig. 5. Our four-year analysis shows that some of these combinations of abuse include heroin and amphetamine, heroin and benzodiazepines, and methamphetamine and cannabis. The number of males who abused two substances was in all cases higher than the number of female abusers. More interestingly, some combinations, such as heroin and cannabis and amphetamine and cannabis were rarely (in 2015) or never abused by females (in 2016, 2017 and 2018). Quantitative information for sex-based abusers of one and two illicit drugs are listed in the Additional files 5 and 6*,* respectively*.*

### Prevalence of illicit drugs in postmortem specimens in Kuwait from 2015 to 2018

Biological samples of deceased individuals were received from the Forensic Medicine Unit of the General Department of Criminal Evidence. The Toxicology Laboratory analyzed the samples to check for the presence of illicit drugs (qualitative analysis). No quantitative investigations were conducted in the present study, and thus, abused drugs were not necessarily the causative agent for the death. The possibility that the deceased subjects may have received prescribed medication cannot be ignored. Thus, data reported herein identify the prevalence of illicit drugs in postmortem specimens from suspected drug-related deaths.

**Fig. 6:**
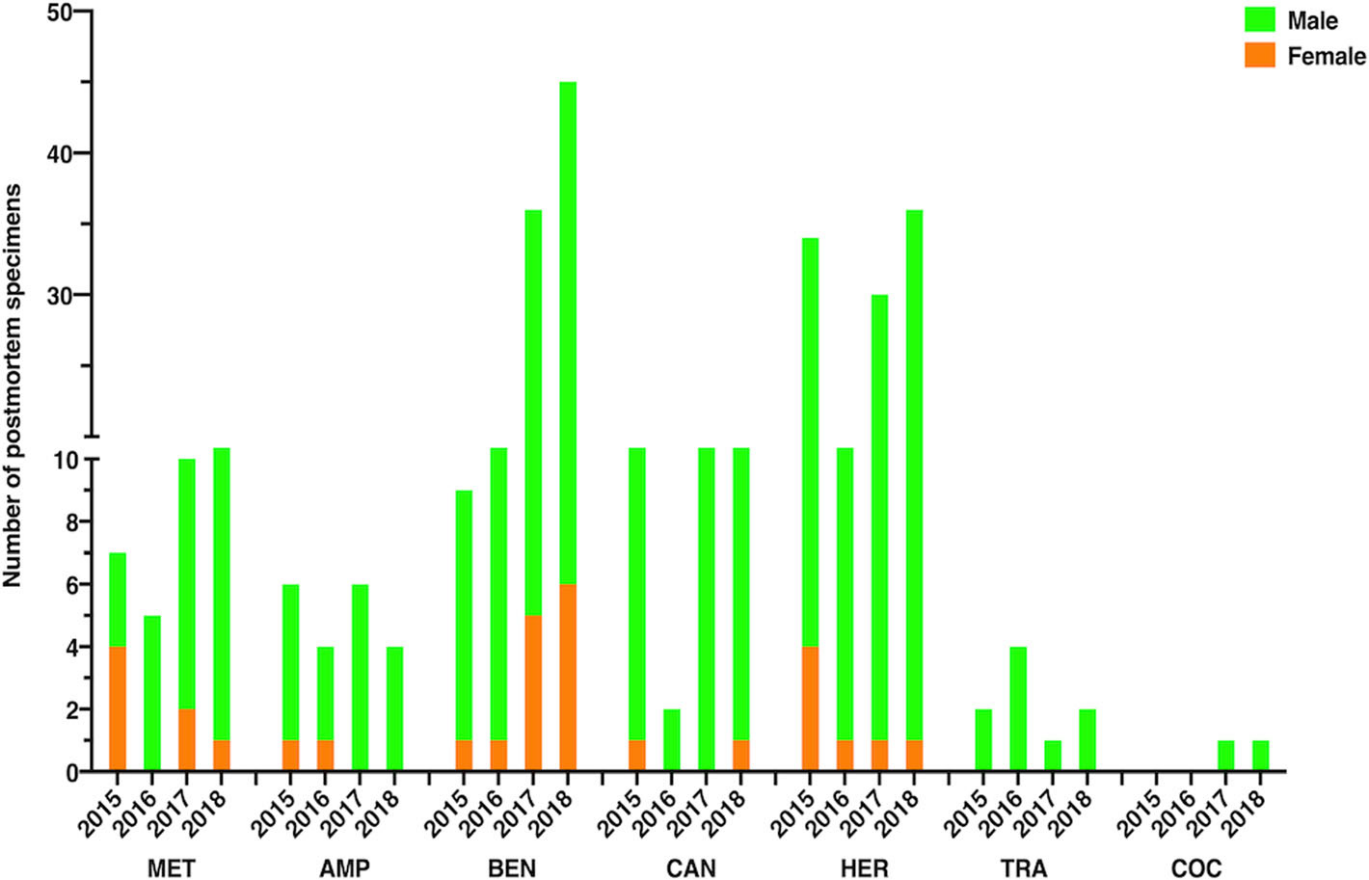
Bar chart showing the number of postmortem specimens with the most identified single drug in Kuwait during 2015–2018 from suspected drug-related deaths. Males and female postmortem samples are differentiated by using different colors (light green for males and orange for females). MET, methamphetamine; AMP, amphetamine; BEN, benzodiazepine; CAN, cannabis; HER, heroin; TRA, tramadol; COC, cocaine. The y-axis is discontinuous due to the wide range of data

Among the identified illicit drugs were methamphetamine, amphetamine, cocaine, heroin, benzodiazepines, tramadol, and cannabis. In the last 3 years, there was an increase in the occurrence of methamphetamine, benzodiazepines, and heroin in the specimens. While benzodiazepine and heroin were the most detected drugs, cocaine and tramadol were the least identified substances. Additionally, cocaine and tramadol were not detected in females’ postmortem specimens (Fig. 6) (Additional file 7).

**Fig. 7:**
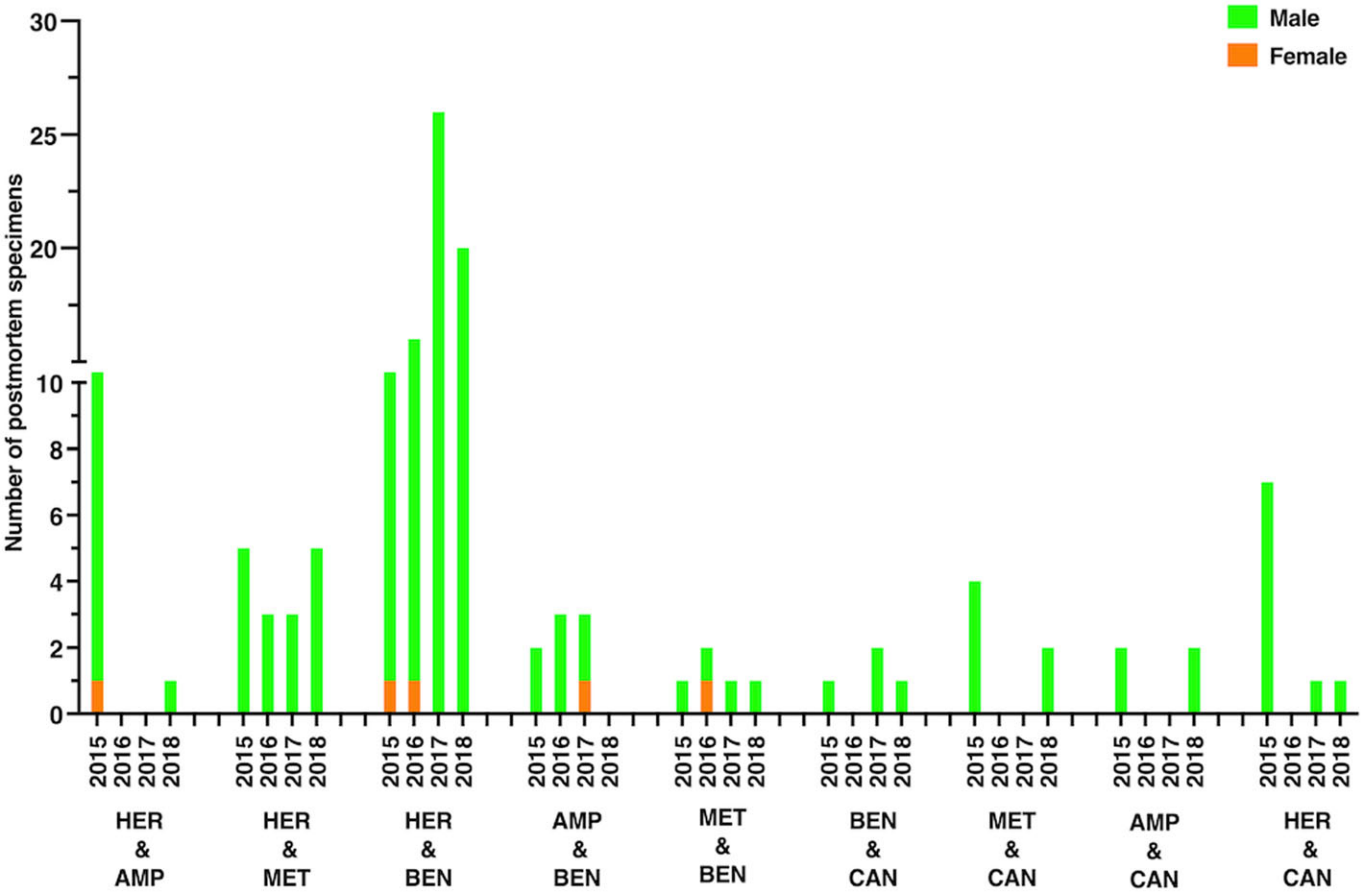
Bar chart showing the number of postmortem specimens with the most identified drug combinations (two-drug cocktail) in Kuwait during 2015–2018 from suspected drug-related deaths. Males and female specimens are differentiated by using different colors (light green for males and orange for females). MET, methamphetamine; AMP, amphetamine; BEN, benzodiazepine; CAN, cannabis; HER, heroin. The y-axis is discontinuous at one point due to the large variations in the data

In addition, we investigated the existence of polydrug, two different illicit drugs (Fig. 7). Our results showed that the two most mixtures were heroin plus benzodiazepines and heroin plus methamphetamine. Some combinations were only identified in some years but not others. Examples of these combinations were heroin and amphetamine (2016 and 2017), amphetamine and benzodiazepines (2018), benzodiazepines and cannabis (2016), methamphetamine and cannabis (2016 and 2017), amphetamine and cannabis (2016 and 2017), and heroin and cannabis (2016). Furthermore, the number females’ specimens that have tested positively for polydrug was negligible (Fig. 7). Details on the identified polydrug in postmortem specimens are reported in Additional file 8.

## Discussion

Drug misuse is a prevalent long-lasting phenomenon affecting individuals in countries all over the world, and the state of Kuwait is not an exception. Therefore, this study was designed to address issues surrounding the abuse of illicit drugs in Kuwait. Our study investigated commonly smuggled types of drugs, the most misused substances based on gender, and the most prevalent drugs in postmortem specimens of suspected drug-related deaths. We found that the most seized substances were cannabis (including marijuana), followed by heroin, opium, and cocaine. Among psychoactive substances, amphetamines (including methamphetamine) were received in large quantities in both powder and pill forms. Other substances seized included benzodiazepines, tramadol, Khat, synthetic cannabinoids, and psilocin. Additionally, amphetamines (including methamphetamine), benzodiazepines, cannabis, and heroin were the most abused substances in Kuwait. Finally, we identified the most common drugs detected in suspected drug-related death (postmortem specimens) with respect to both gender and substance combinations.

### Trafficking and Marketing of Illicit Drugs in Kuwait

The initial objective of our study was to determine the different types of narcotic drugs and psychotropic substances that were seized and consumed in Kuwait from 2015 to 2018. In addition, the study aimed to identify mortalities associated with the consumption of these illicit substances. Our analysis reports the different types and quantities of illicit drugs that were seized in Kuwait from 2015 to 2018 (Figs. 1 and 2). The variety of drugs seized is primarily associated with the geographical situation of Kuwait relative to major drug-producing countries, including Afghanistan, Pakistan, Iraq, and Iran [22]. However, the variety of drugs seized may also reflect the rapid financial development of Kuwait; its economic prosperity may have made the country an excellent target for the illicit drugs market. The data revealed annual fluctuations in the quantities of the same seized substances. These variations may be associated with the changes in 1) flow of the illicit drugs from the country of origin; 2) smuggling strategies (methods and routes); and 3) activities of Kuwaiti law enforcement.

Cannabis and marijuana were the most often seized substances among narcotic drugs (i.e., opium, heroin, and cocaine). These data are consistent with the International Narcotics Control Board (INCB) report (2018), which stated that cannabis trafficking into or through Kuwait increased by 233% from 2016 to 2017 [23].

Afghanistan supplies the gulf countries with many illicit drugs, including opium, heroin, and cannabis; these pass through the Iranian docks of Chabahar and Bandar Abbas. According to the INCB report [23], there are indications that Iraq is becoming a key player for illicit drug cultivation and production, including heroin, opium poppy, and cannabis.

Another important finding was that low quantities of cocaine were received during the 4 years of the study (Fig. 2). These findings agree with a previous DEA report, which revealed a weak association between the Arab world and the production or trafficking of cocaine. Nevertheless, there is some evidence for an increase in seizures of cocaine in the Middle East that are destined for distribution in Western Europe. The Kuwaiti market did show noticeable quantities of Khat, and this may have been smuggled from Ethiopia and Yemen. While Ethiopian Khat is smuggled by Ethiopian workers via air routes, Khat from Yemen is smuggled to Kuwait via land routes through the borders with Saudi Arabia. Even though Khat use is uncommon among Kuwaiti locals, there may be some influence from neighboring countries with high Khat consumption, including Yemen, Oman, and Saudi Arabia [24].

The most obvious finding to emerge from our study is that illicit use of amphetamine-type stimulants (ATS), including methamphetamine and amphetamine, is highly prevalent in Kuwait (Figs. 2 and 3). The NPL usually receives amphetamine as a component of *Captagon* pills smuggled in from Iraq. In November 2017, 599 bags of Captagon were seized by law enforcement officers in the Basra region of Iraq, near the Kuwaiti border. Our data is consistent with the European Union Institute for Security Studies (EUISS) November 2017 report [10], which declared that the Gulf countries are facing an amphetamine-type stimulant (ATS) addiction crisis. The report showed that abuse of these stimulants accounted for more than 62% of the admissions into Saudi rehab facilities. According to the report, the increase in ATS addiction in the MENA area could be attributed to the impact of modernization, and exposure to modern Western cultures and lifestyles.

The 2016 blanket ban of Synthetic cannabinoids in Kuwait following the footsteps of the UK Psychoactive Substance Act [25], resulted in the changes observed in the number of seizures for such drug in Kuwait. Additional surveillance is required to see how well did the change in seizures correspond with the number of emergency department visits and rehab admissions.

The illegal use of medically prescribed drugs is a worldwide health concern, and our data confirm that the state of Kuwait is not an exception. Several different psychoactive substances were seized by Kuwaiti law enforcement, including tramadol and drugs of the benzodiazepine family. Our data show that confirmed users of psychoactive substances also tested positive for other illicit drugs (i.e. heroin, cannabis, and methamphetamine). Thus, an assumption can be made that the abuse of these psychoactive drugs was associated with behavioral addictions rather than for medical purposes. These substances were abused for pleasure purposes to induce an altered state of consciousness through modifying the perceptions, feelings, and emotions of the user.

Tramadol is a synthetic opioid analgesic usually prescribed to manage moderate to severe levels of pain. Tramadol misuse is a matter of genuine concern in the Gulf countries, as an overdose is associated with significant morbidity and mortality [6]. The abuse of tramadol in the United Arab Emirates has been previously reported [26]. According to the INCB [23], tramadol is used (non-medically) as a mood enhancer, to enhance sexual stamina, or to boost energy during physical activities. However, long-term use of tramadol results in psychological and physical dependence, increasing the risk of overdose [23]. In this study, we showed that large quantities of tramadol have been seized, and it is assumed that these are to be used illegally for non-medical purposes (Fig. 1). Our results show that the quantity seized (in pill form) were as follows: 40,050 in 2015, 7,460,319 in 2016, 10,150 in 2017 and 42,213 in 2018.

Tranquillizers of the benzodiazepine family were among the most popular substances of abuse seized from the Kuwaiti market. These included clonazepam, diazepam, flunitrazepam, alprazolam, and bromazepam (Fig. 3). Our data showed that the classes of benzodiazepines most commonly seized were clonazepam, diazepam, and alprazolam (Fig. 3). Benzodiazepines slow down bodily functions by enhancing the influence of the inhibitory neurotransmitter γ-aminobutyric acid (GABA), which interacts with GABA_A_ receptors to increase their sedative and anxiolytic actions. A previous survey reported that benzodiazepines were among the drugs most abused by youth in Saudi Arabia [27]. Benzodiazepines can be obtained in Kuwait either as smuggled substances from countries such as Iran, Saudi Arabia, the United Arab Emirates, and Egypt, or through illegal purchase from pharmacies without a prescription (or sometimes with a falsified prescription). In Kuwait, Strict laws need to be implemented regarding the prescription of drugs in general, and especially drugs with abuse potential, such laws should have a positive outcome on the number of hospitalisations, number of rehab cases and other clinical implications.

### Abuse of illicit drugs in Kuwait compared to neighboring countries

The misuse of intravenous illicit drugs has been reported in all MENA countries. Several studies estimate that the number of individuals that are injecting drugs in the region is 349,500–437,000. While 96.2% of abusers were reported to use opioids as their primary drug, 14.2% were abusing stimulants [28, 29]. In this study, we investigated the number of illicit drug abusers in Kuwait from 2015 to 2018. Most of the abusers used methamphetamine, cannabis, amphetamine, or heroin. The abuse of benzodiazepine, tramadol, and cocaine was less common (Fig. 4). The use of amphetamine-type stimulants has been well established in the Middle East [30].

Our study also highlighted gender-based differences among substance abusers (Fig. 4). In general, the number of female abusers was much lower than the number of male abusers (Fig. 4). These differences can be explained in part by male dominance in the Kuwaiti culture. In addition, previous studies have shown that males abuse almost all kinds of illicit drugs and that they do so much more than females [17]. This extreme abuse by males increases their chances of a visit to the emergency department and of overdose associated mortality [17]. However, women are just as likely as men to develop a substance use disorder [31]. Moreover, studies suggest that females are more susceptible to craving and relapse, two critical phases of the addiction cycle [32–37].

Some abusers were addicted to combinations of multiple drugs, usually a combination of two substances (Fig. 5), but sometimes more (data not shown). Some combinations were more prevalent in a particular year than others. For instance, while the combination of methamphetamine and cannabis was commonplace in 2015, the popularity of this mixture changed dramatically in subsequent years. This finding is consistent with a report that cannabis is the most common secondary illicit substance for methamphetamine users [38]. The number of individuals co-abusing heroin and benzodiazepines remained almost the same throughout the 4 years. This observation is in agreement with that in a study by Jones et al. reported that co-abuse of opioids and benzodiazepines is ubiquitous worldwide [39]. According to Jones et al., co-abuse of benzodiazepine and opioids is primarily for recreational purposes, to enhance the opioid intoxication or “high” and to enable doses that exceed the therapeutic range [39]. A second explanation for the use of benzodiazepines is self-medication; i.e., the treatment of anxiety, mania, or insomnia. Combinations of multiple drugs were resolved and identified using GC/MS and LC/MS. Other analytical techniques such as FT-IR and UV techniques are not suitable for the analysis of such complex samples.

### Prevalence of illicit drugs in postmortem specimens in Kuwait from 2015 to 2018

Drug misuse induces premature deaths [3]. In the current study, we investigated common drugs (single and two-drugs cocktail) that detected in postmortem samples from potential drug-related deaths. These drugs were methamphetamine, amphetamine, benzodiazepines, cannabis, heroin, tramadol, and cocaine (Figs. 6 and 7). Our data showed that the most common drugs were heroin, benzodiazepines, and methamphetamine (Fig. 6). In contrast, tramadol and cocaine were the least detected drugs (Fig. 6). The infrequent detection of cocaine may be due to the negligible amount of the drug in the Kuwaiti market for illegal.

Furthermore, different two-drugs cocktails were identified. Heroin-benzodiazepines cocktail is the most common two-drug combination detected in postmortem specimens. Other combinations were also identified, including cannabis-benzodiazepines and cannabis-amphetamines (including methamphetamine) (Fig. 7). The qualitative detection of these substances during postmortem analysis is inadequate to verify drug misuse as the primary cause of death. Drug-related deaths are complex to confirm and require thorough investigative information, including quantitative drug analysis, medical history, crime-scene details, and physiological findings of the autopsy [40]. Thus, our reported data should be interpreted with caution in terms of drug-related mortality due to the qualitative nature of the toxicological investigation.

### Limitations of the study

As with the majority of studies, the design of the current study is subject to limitations. The most critical flaw lies in the fact that no quantitative analysis was conducted, and the results reported herein were obtained qualitatively. Consequently, we were unable to generalize from some of the research findings with any confidence, including those of drug-induced mortality among drug abusers. Fatality association with drug overdose cannot be established based on qualitative analysis of the drug profile. Moreover, the possibility that the deceased subject used some of the identified drugs for medical purposes cannot be excluded (in which case these drugs may not be a causative agent for the death). Similarly, no quantitative confirmation was used for reporting drug concentrations in the biological samples obtained from living subjects. Only a simple “positive or negative” result was provided in the final report for confirmation of drug abuse. Thus, further research should be undertaken, including a quantitative approach, to provide a comprehensive analysis of illicit drug abuse. Only then can we tackle the drug misuse crisis in Kuwait and actively promote our society as free of illicit substance addiction. An additional source of weakness is our generalization to drug families during drug abuse analysis of biological samples (without specifying the exact derivative). For example, while the existence of benzodiazepines in the samples may be confirmed, identification of the exact derivative was not performed.

## Conclusions

In this paper we presented an MS based analytical platform that analysed 6220 drug samples and 17,755 toxicological samples between the years 2015–2018. The data revealed that cannabis was the most seized illicit drug, while methamphetamine was the most abused. According to postmortem toxicological analysis, benzodiazepine and heroin were the most detected single drug. Also, the heroin-benzodiazepine multiple drug cocktail was the predominant identified combination. This study provides valuable data to local and international drug analyst, law enforcement and health officials. Our findings suggest that there is a growing need to conduct larger scale studies to implement new strategies, policies, and interventions for positive outcomes in populations affected by illicit drugs.

## Supplementary Information

**Additional file 1.** Photographic images of seized drugs.

**Additional file 2.** Narcotic substances received by the NPL of Kuwait (2015–2018).

**Additional file 3.** Psychoactive substances received by the NPL of Kuwait (2015–2018).

**Additional file 4.** Psychoactive substances (pills) received by the NPL of Kuwait (2015–2018).

**Additional file 5.** Number of abusers of one illicit substance (2015–2018).

**Additional file 6.** Number of abusers of two illicit substances (2015–2018).

**Additional file 7.** Prevalence of one illicit substance identified in postmortem specimens (2015–2018).

**Additional file 8.** Prevalence of two illicit substances identified in postmortem specimens (2015–2018).

## Data Availability

The datasets used and/or analysed during the current study available from the corresponding author on reasonable request.

## Abbreviations

AACC: American Association for Clinical Chemistry
AGC: Automatic Gain Control
ATS: Amphetamine-type stimulant
CCD: Charged coupled device
CDS: Chromatography Data System
DEA: Drug Enforcement Agency
DOA: Drug of Abuse
EI: Electron ionization
EPI: Enhanced product ion
FMU: Forensic Medicine Unit
FTL: Forensic Toxicology Laboratory
GC: Gas chromatography
HRAM: High-resolution, accurate-mass
IDA: Information-dependent acquisition
MENA: Middle East and North Africa
MENAHRA: Middle East and North Africa Harm Reduction Association
MRM: Multiple reaction monitoring
MS: Mass spectrometry
NMR: Nuclear magnetic resonance
NPL: Narcotics and Psychotropic Laboratory
ONDCP: Office of National Drug Control Policy
SPE: Solid phase extraction
TLC: Thin layer chromatography
UNODC: United Nations Office on Drugs and Crime
UV: Ultraviolet-visible
