# Supplementary material for: Snapshot of narcotic drugs and psychoactive substances in Kuwait: analysis of illicit drugs use in Kuwait from 2015 to 2018
Source: BMC Public Health. 2021 Apr 7;21:671. doi: 10.1186/s12889-021-10705-z (PMC8028837; doi:10.1186/s12889-021-10705-z)

**Additional file 1.** Photographic images of seized drugs. Representative images show examples of seized drugs (natural and synthetic) in Kuwait (2015*–*2018). Pill and capsule forms are shown in front and rear view. (A-C) examples of amphetamine-type stimulants (ATS) in both pill and crystal forms and in different colors: (A) methamphetamine; (B) Captagon; (C) methamphetamine crystals; (D) tramadol in different forms (pill and capsule) and colors; (E, F) examples of benzothiazine drugs: (E) clonazepam; (F) alprazolam; (G) ecstasy [3,4-methylenedioxy-methamphetamine (MDMA)], a recreational pill obtained in different shape and colors; (H) cannabis in dried flower and oil-like forms. The photographs represent actual seized items that processed by the Narcotic and Psychotropic Laboratory. The images were taken by the Author M. A. using a digital camera (Canon PowerShot SX50 HS, Japan).


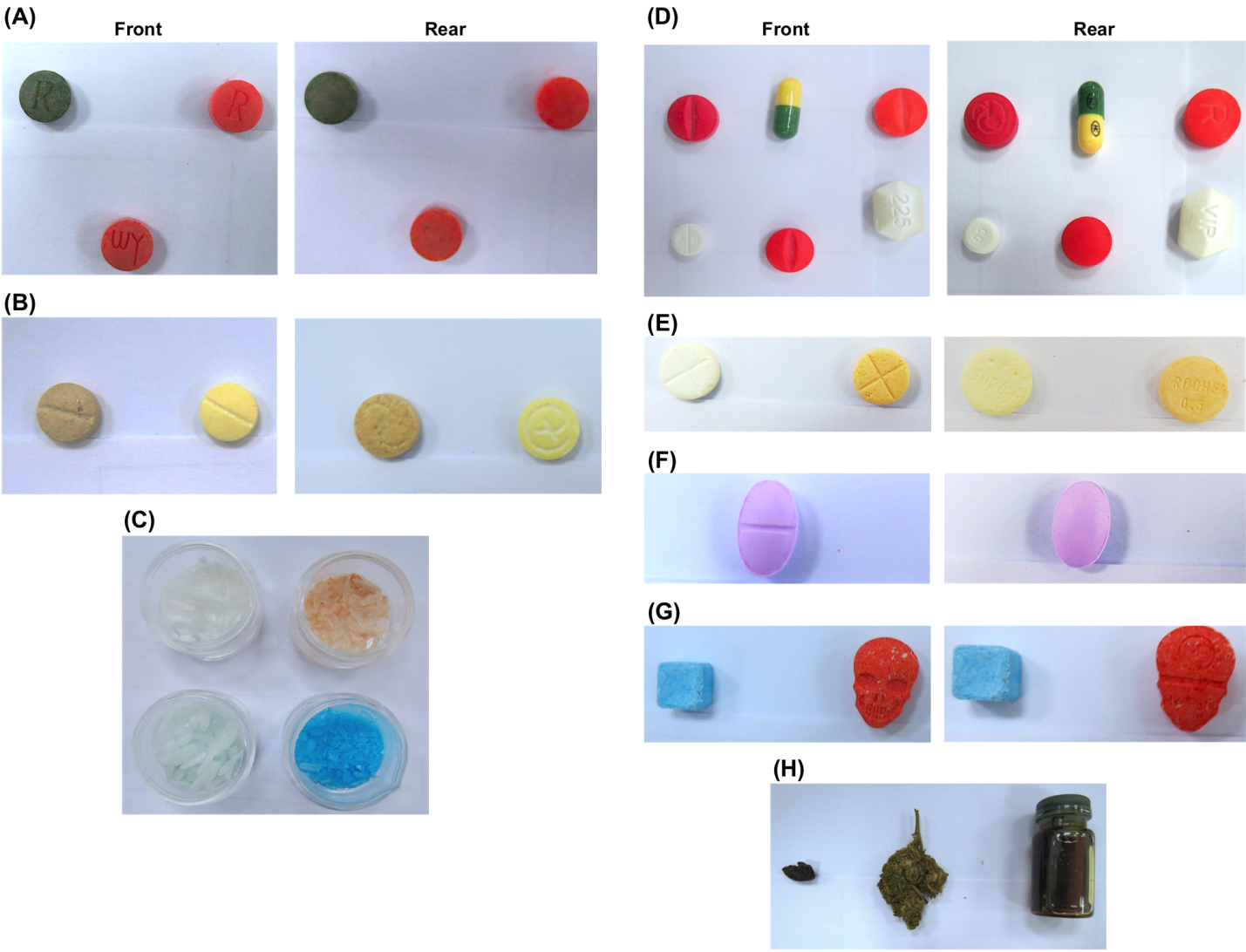

Supplement: Supplementary file 1 — Additional file 1. Photographic images of seized drugs. [file 12889_2021_10705_MOESM1_ESM.docx]
