# Supplementary material for: Snapshot of narcotic drugs and psychoactive substances in Kuwait: analysis of illicit drugs use in Kuwait from 2015 to 2018
Source: BMC Public Health. 2021 Apr 7;21:671. doi: 10.1186/s12889-021-10705-z (PMC8028837; doi:10.1186/s12889-021-10705-z)
Supplement: Supplementary file 2 — Additional file 2. Narcotic substances received by the NPL of Kuwait (2015–2018). [file 12889_2021_10705_MOESM2_ESM.docx]

**Additional file 2.** Narcotic substances received by the NPL of Kuwait (2015–2018)

| Marijuana | Cannabis | Heroin | Opium | Cocaine | Year |
| --- | --- | --- | --- | --- | --- |
| 95,681.730 | 652,759.480 | 47,567.860 | 10,221.460 | 494.890 | 2015 |
| 116,472.660 | 632,052.630 | 21,272.740 | 5,677.320 | 3.080 | 2016 |
| 14,614.133 | 43,781,809.500 | 26,485.288 | 1,868.060 | 105.840 | 2017 |
| 39,809.860 | 3,234,105.988 | 25,960.135 | 2,168.830 | 1,240.600 | 2018 |
