# Supplementary material for: Snapshot of narcotic drugs and psychoactive substances in Kuwait: analysis of illicit drugs use in Kuwait from 2015 to 2018
Source: BMC Public Health. 2021 Apr 7;21:671. doi: 10.1186/s12889-021-10705-z (PMC8028837; doi:10.1186/s12889-021-10705-z)
Supplement: Supplementary file 3 — Additional file 3. Psychoactive substances received by the NPL of Kuwait (2015–2018). [file 12889_2021_10705_MOESM3_ESM.docx]

**Additional file 3.** Psychoactive substances received by the NPL of Kuwait (2015–2018)

| PSY | KHA | SYN CAN | MET | Year |
| --- | --- | --- | --- | --- |
| 7,549.320 | 50,698.62 | 32,484.670 | 29,732.920 | 2015 |
| 16,338.500 | 19,885.200 | 1,420.300 | 24,826.770 | 2016 |
| - | 11,109.500 | 2,003,897.784 | 21,376.662 | 2017 |
| - | 83,183.29 | 25,561.66 | 60,082.84 | 2018 |

MET, methamphetamine; SYN CAN, synthetic cannabinoids; KHA, Khat; PSY, psilocybin mushrooms
