# Supplementary material for: Snapshot of narcotic drugs and psychoactive substances in Kuwait: analysis of illicit drugs use in Kuwait from 2015 to 2018
Source: BMC Public Health. 2021 Apr 7;21:671. doi: 10.1186/s12889-021-10705-z (PMC8028837; doi:10.1186/s12889-021-10705-z)
Supplement: Supplementary file 4 — Additional file 4. Psychoactive substances (pills) received by the NPL of Kuwait (2015–2018). [file 12889_2021_10705_MOESM4_ESM.docx]

**Additional file 4.** Psychoactive substances (pills) received by the NPL of Kuwait (2015–2018)

| TRA | BRO | ALP | FLU | DIA | COL | AMP | MET | Year |
| --- | --- | --- | --- | --- | --- | --- | --- | --- |
| 49,050 | 634 | 1093 | 405 | 2,285 | 21,839 | 28,376. | 6,433. | 2015 |
| 7,460,819 | 6,836 | 1,230,520 | 130 | 128,238 | 556,816 | 2,598,398 | 1,209 | 2016 |
| 10,150 | 168 | 2,676 | 963.5 | 474 | 1,145.5 | 26,262 | 1,190 | 2017 |
| 42,213 | 479 | 673.50 | 193.50 | 1,007 | 125,291.54 | 24,510.65 | 10,711.98 | 2018 |

MET, methamphetamine; AMP, amphetamine; CLO, clonazepam; DIA, diazepam; FLU, flunitrazepam; ALP, alprazolam; BRO, bromazepam; TRA, tramadol
