# Supplementary material for: Snapshot of narcotic drugs and psychoactive substances in Kuwait: analysis of illicit drugs use in Kuwait from 2015 to 2018
Source: BMC Public Health. 2021 Apr 7;21:671. doi: 10.1186/s12889-021-10705-z (PMC8028837; doi:10.1186/s12889-021-10705-z)
Supplement: Supplementary file 5 — Additional file 5. Number of abusers of one illicit substance (2015–2018). [file 12889_2021_10705_MOESM5_ESM.docx]

**Additional file 5.** Number of abusers of one illicit substance (2015–2018)

| COC | | TRA | | HER | | CAN | | BEN | | AMP | | MET | | Year |
| --- | --- | --- | --- | --- | --- | --- | --- | --- | --- | --- | --- | --- | --- | --- |
| F | M | F | M | F | M | F | M | F | M | F | M | F | M |  |
| 0 | 0 | 0 | 2 | 10 | 108 | 8 | 389 | 3 | 59 | 3 | 175 | 36 | 560 | 2015 |
| 0 | 0 | 0 | 8 | 4 | 86 | 5 | 142 | 3 | 58 | 9 | 210 | 17 | 411 | 2016 |
| 0 | 1 | 0 | 5 | 7 | 129 | 4 | 187 | 9 | 85 | 8 | 129 | 4 | 190 | 2017 |
| 0 | 1 | 0 | 11 | 3 | 117 | 6 | 84 | 4 | 84 | 4 | 84 | 15 | 284 | 2018 |

MET, methamphetamine; AMP, amphetamine; BEN, benzodiazepine; CAN, cannabis; HER, heroin; TRA, tramadol; COC, cocaine. (M, male; F, female)
