# Supplementary material for: Snapshot of narcotic drugs and psychoactive substances in Kuwait: analysis of illicit drugs use in Kuwait from 2015 to 2018
Source: BMC Public Health. 2021 Apr 7;21:671. doi: 10.1186/s12889-021-10705-z (PMC8028837; doi:10.1186/s12889-021-10705-z)
Supplement: Supplementary file 6 — Additional file 6. Number of abusers of two illicit substances (2015–2018). [file 12889_2021_10705_MOESM6_ESM.docx]

**Additional file 6.** Number of abusers of two illicit substances (2015–2018)

| HER  &  CAN | | AMP  &  CAN | | MET  &  CAN | | BEN  &  CAN | | MET  &  BEN | | AMP  &  BEN | | HER  &  BEN | | HER  &  MET | | HER  &  AMP | | Year |
| --- | --- | --- | --- | --- | --- | --- | --- | --- | --- | --- | --- | --- | --- | --- | --- | --- | --- | --- |
| F | M | F | M | F | M | F | M | F | M | F | M | F | M | F | M | F | M |  |
| 1 | 32 | 2 | 107 | 10 | 320 | 1 | 12 | 0 | 9 | 1 | 17 | 8 | 67 | 1 | 20 | 4 | 92 | 2015 |
| 0 | 3 | 0 | 15 | 1 | 42 | 0 | 10 | 3 | 38 | 2 | 23 | 5 | 53 | 1 | 25 | 0 | 13 | 2016 |
| 0 | 6 | 0 | 19 | 1 | 30 | 0 | 11 | 0 | 12 | 2 | 14 | 2 | 58 | 1 | 28 | 1 | 7 | 2017 |
| 0 | 9 | 0 | 37 | 0 | 2 | 0 | 3 | 0 | 3 | 0 | 1 | 0 | 33 | 2 | 36 | 0 | 2 | 2018 |

MET, methamphetamine; AMP, amphetamine; BEN, benzodiazepine; CAN, cannabis; HER, heroin. (M, male; F, female)
