# Supplementary material for: Snapshot of narcotic drugs and psychoactive substances in Kuwait: analysis of illicit drugs use in Kuwait from 2015 to 2018
Source: BMC Public Health. 2021 Apr 7;21:671. doi: 10.1186/s12889-021-10705-z (PMC8028837; doi:10.1186/s12889-021-10705-z)
Supplement: Supplementary file 7 — Additional file 7. Prevalence of one illicit substance identified in postmortem specimens (2015–2018). [file 12889_2021_10705_MOESM7_ESM.docx]

**Additional file 7.** Prevalence of one illicit substance identified in postmortem specimens (2015–2018)

| COC | | TRA | | HER | | CAN | | BEN | | AMP | | MET | | Year |
| --- | --- | --- | --- | --- | --- | --- | --- | --- | --- | --- | --- | --- | --- | --- |
| F | M | F | M | F | M | F | M | F | M | F | M | F | M |  |
| 0 | 0 | 0 | 2 | 4 | 30 | 1 | 14 | 1 | 8 | 1 | 5 | 4 | 3 | 2015 |
| 0 | 0 | 0 | 4 | 1 | 16 | 0 | 2 | 1 | 11 | 1 | 3 | 0 | 5 | 2016 |
| 0 | 1 | 0 | 1 | 1 | 29 | 0 | 11 | 5 | 31 | 0 | 6 | 2 | 8 | 2017 |
| 0 | 1 | 0 | 2 | 1 | 35 | 1 | 17 | 6 | 39 | 0 | 4 | 1 | 13 | 2018 |

MET, methamphetamine; AMP, amphetamine; BEN, benzodiazepine; CAN, cannabis; HER, heroin; TRA, tramadol; COC, cocaine. (M, male; F=female)
