# Supplementary material for: Snapshot of narcotic drugs and psychoactive substances in Kuwait: analysis of illicit drugs use in Kuwait from 2015 to 2018
Source: BMC Public Health. 2021 Apr 7;21:671. doi: 10.1186/s12889-021-10705-z (PMC8028837; doi:10.1186/s12889-021-10705-z)
Supplement: Supplementary file 8 — Additional file 8. Prevalence of two illicit substances identified in postmortem specimens (2015–2018). [file 12889_2021_10705_MOESM8_ESM.docx]

**Additional file 8.** Prevalence of two illicit substances identified in postmortem specimens (2015–2018)

| HER  &  CAN | | AMP  &  CAN | | MET  &  CAN | | BEN  &  CAN | | MET  &  BEN | | AMP  &  BEN | | HER  &  BEN | | HER  &  MET | | HER  &  AMP | | Year |
| --- | --- | --- | --- | --- | --- | --- | --- | --- | --- | --- | --- | --- | --- | --- | --- | --- | --- | --- |
| F | M | F | M | F | M | F | M | F | M | F | M | F | M | F | M | F | M |  |
| 0 | 7 | 0 | 2 | 0 | 4 | 0 | 1 | 0 | 1 | 0 | 2 | 1 | 13 | 0 | 5 | 1 | 10 | 2015 |
| 0 | 0 | 0 | 0 | 0 | 0 | 0 | 0 | 1 | 1 | 0 | 3 | 1 | 15 | 0 | 3 | 0 | 0 | 2016 |
| 0 | 1 | 0 | 0 | 0 | 0 | 0 | 2 | 0 | 1 | 1 | 2 | 0 | 26 | 0 | 3 | 0 | 0 | 2017 |
| 0 | 1 | 0 | 2 | 0 | 2 | 0 | 1 | 0 | 1 | 0 | 0 | 0 | 20 | 0 | 5 | 0 | 1 | 2018 |

MET = methamphetamine, AMP = amphetamine, BEN = benzodiazepine, CAN = cannabis, HER = heroin. (M, male; F, female)
